# Evaluation of the warming sensation, acceptability, and local tolerability of an acetylcysteine oral solution containing the flavoring agent IFF flavor 316282 in the treatment of productive cough 

**DOI:** 10.5414/CP203125

**Published:** 2018-02-16

**Authors:** Frank Donath, Anna Grinienko, Pascal Mallefet, Michel Jean-Pierre Ozun, Lucy Shneyer

**Affiliations:** 1 SocraTec R&D GmbH, Erfurt, Germany,; 2 Clinical Development and Medical Affairs, GSK Consumer Healthcare, Warren, NJ, USA,; 3 Clinical Development and Medical Affairs, GSK Consumer Healthcare, Nyon, Switzerland, and; 4 Shneyer Statistics LLC, Denville, NJ, USA

**Keywords:** acetylcysteine, expectorants, flavoring agents, oral solution, warming sensation

## Abstract

Objective: This open-label study sought to evaluate the warming sensation produced by IFF flavor 316282 in an acetylcysteine oral solution in subjects with productive cough. Materials: 2% acetylcysteine oral solution (200 mg per 10 mL) containing IFF flavor 316282. Methods: Subjects (N = 57; mean age 38.7 years; 58% female) with a productive cough lasting < 7 days and rated as mild to moderate in severity received 10 mL of study product. Warming sensation intensity was assessed using a 100-mm visual analog scale, its onset and duration using stopwatches, its acceptability using a 9-point scale (from “dislike extremely” to “like extremely”) and the taste, texture, and overall acceptability of the solution using 5-point scales (from “unacceptable” to “excellent”). Results: 53 (93.0%) subjects perceived a warming sensation within 10 minutes of swallowing the solution; median onset was ~ 14 seconds, and median duration was ~ 2.8 minutes. Warming sensation intensity increased from baseline by a mean of 29.2 mm when evaluated 60 seconds after ingestion. 30 subjects (52.6%) thought the warming sensation was “just about right”; 25 (43.9%) considered it “too weak” or “much too weak.” Most subjects had positive overall ratings (“fair,” “good,” or “excellent”) of the taste (79.0%), texture (96.5%), and solution (91.2%). No treatment-emergent adverse events were reported, and no evidence of oral mucosal irritation was found. Conclusion: The addition of IFF flavor 316282 to a 2% acetylcysteine oral solution produced a warming sensation with rapid onset and relatively short duration, which the majority of subjects found acceptable.

## Introduction 

Acetylcysteine is a mucolytic agent with established efficacy and tolerability in acute and chronic bronchopulmonary diseases accompanied by impaired formation and transport of mucus, including bronchitis and the common cold, and as an antidote for acetaminophen overdose [1, 2]. Acetylcysteine is the acetylated variant of the amino acid L-cysteine, and serves as an excellent source of exposed sulfhydryl groups that promote hydrolysis of disulfide bonds in respiratory mucin oligomers and thereby reduce mucus viscosity [3, 4]. The activity of acetylcysteine may also derive from other mechanisms, including antioxidant properties and impairment of bacterial adhesion to epithelial cells [3]. 

A new oral solution formulation containing acetylcysteine (200 mg per 10 mL) has been developed for subjects with bronchitis associated with the common cold and characterized by productive cough. By reducing the viscosity of mucus secretions, the oral solution aids in the elimination of mucus from the respiratory tract by expectoration, thereby facilitating productive cough. The formulation contains a flavoring agent (flavor 316282; International Flavors & Fragrances (IFF), New York, NY, USA) in a concentration of 0.15% weight/volume, which has been previously shown to produce a warming sensation in the throat [5, 6]. The present study was designed to assess the warming sensation, acceptability, tolerability, and safety of the new oral solution formulation containing acetylcysteine 200 mg per 10 mL with IFF flavor 316282 in subjects with a productive cough caused by an upper respiratory tract infection (URTI). 

## Materials and methods 

### Materials 

An oral solution containing IFF flavor 316282 and 2% acetylcysteine (200 mg per 10 mL) was stored in a 100-mL amber bottle. The solution was administered at room temperature (below 25 °C) by study staff in a dose of 10 mL using an oral syringe. 

### Study design 

This single-center, single-treatment arm, open-label study (ClinicalTrials.gov identifier NCT02822287) was performed at the Clinical Pharmacology Unit of SocraTec R&D GmbH (Erfurt, Germany) between February 23 and March 22, 2016 and was sponsored by Novartis Consumer Health, a GlaxoSmithKline Consumer Healthcare company (NCH-GSK; Nyon, Switzerland). The study was conducted in accordance with the ethical principles of the Declaration of Helsinki and in compliance with the International Council on Harmonisation Guideline for Good Clinical Practice and other applicable regulations. An Independent Ethics Committee approved the study protocol, and all subjects provided written informed consent before participating. 

Subjects were screened on the study day and, if eligible, underwent a washout period of 0.5 to 8 hours, completed predose assessments, received supervised dosing of the study product, and participated in a 1-hour postdose evaluation. During the predose period, study staff administered 10 mL of water at room temperature, using an oral syringe, to establish warming sensation at baseline. For evaluation of the study product, study staff administered 10 mL of the 2% acetylcysteine oral solution containing IFF flavor 316282 over a 1-minute period. The postdose evaluation period began once the subject successfully swallowed the entire dose of study medication; those who failed to consume all or part of the dose were to be discontinued from the study. 

### Subjects 

Male and female subjects aged 12 years or older were eligible if they had a productive cough due to URTI lasting < 7 days in duration that was rated as mild to moderate in severity immediately prior to dosing based on a 4-point ordinal scale (not present, mild, moderate, or severe). Adolescents were eligible only after a review of data from the first 18 adult subjects confirmed the tolerability and warming sensation of the study medication. Subjects with a severe sore throat or temperature ≥ 39.4 °C prior to dosing were excluded. Other exclusion criteria were asthma, gastroduodenal ulcers, severe renal impairment, hypertension, hyperthyroidism, diabetes, heart disease, and current smoking (≥ 10 cigarettes per day or equivalent). Pregnant and lactating women were also excluded, as were women of child-bearing potential who were not using an effective method of contraception. 

Use of caffeinated or hot beverages within 1 hour prior to dosing; certain antibiotics within 2 hours prior to dosing; any inhalers, throat lozenges or sprays, products with demulcent or local anesthetic properties, products containing menthol, peppermint, or spearmint, acetaminophen or a nonsteroidal anti-inflammatory drug, or oral/nasal decongestants within 6 hours prior to dosing; antitussives or anticholinergic agents within 24 hours prior to dosing; and herbal medications or antihistamines within 48 hours prior to dosing was prohibited. Subjects were not allowed to gargle, eat, chew gum, or use any breath freshener from the start of screening until completion of the study. If required, subjects could sip up to 200 mL of noncarbonated water during the period from 1 hour before dosing until completion of the study, except during the 10-minute period immediately after ingestion of study medication. 

### Assessments 

Subjects evaluated the intensity, onset, duration, and acceptability of the warming sensation of the study medication and provided their overall opinion of the warming sensation and of the oral product. The intensity of the warming sensation mainly in the throat and at the back of the mouth was evaluated after ingestion of water during the predose period (baseline) and 60 to 75 seconds after ingestion of the study medication. Subjects were asked to swallow and indicate the intensity of the warming sensation on a 100-mm visual analog scale (VAS) from 0 (no warming sensation) to 100 (strongest possible warming sensation). To assess onset and duration of the warming sensation, 2 stopwatches were started when the subjects swallowed the study medication, with the faces of the stopwatches totally covered by labels with the wording “start of warming” and “end of warming,” respectively. Subjects were instructed to stop the first watch when they noticed the onset of a warming sensation and the second watch when they no longer felt the warming sensation. Subjects evaluated the acceptability of the warming sensation at 10 minutes after dosing by answering the question “*How strong is the warming sensation level you have experienced for this product?*” using a 5-point ordinal scale, where 1 = much too weak, 2 = too weak (not warming enough), 3 = just about right (pleasant warming), 4 = too strong (too warming), and 5 = much too strong. 

Subjects provided their overall acceptability opinions of the warming sensation at 10 minutes after dosing and of the oral solution at 1 hour. For the former, subjects answered the question “*How do you like the warming sensation you have experienced for this product?*” using a 9-point ordinal scale ranging from 1 (dislike extremely) to 9 (like extremely). For the overall opinion of the oral solution, subjects answered 3 questions (*How would you rate the taste of the oral solution you took? How would you rate the texture of the oral solution you took? How would you rate overall the oral solution?*) using a 5-point ordinal scale, where 0 = unacceptable, 1 = poor, 2 = fair, 3 = good, and 4 = excellent. 

Safety and tolerability assessments included adverse-event monitoring throughout the study; vital sign measurements at screening, predose, and at the end of the study visit; and physical examinations at screening and at the end of the study. In addition, a thorough oropharyngeal examination was made using a lighting device to assess any signs of lesions or irritations on the oral mucosa before and after administration of the study product. 

### Statistical analysis 

Based on prior sensory panel tests and local acceptability studies using similar oral solutions containing IFF 316282, a sample size of 70 subjects was required to obtain 56 evaluable subjects completing the 1-hour assessment period, assuming a 20% dropout rate. The intensity and acceptability of the warming sensation, and the acceptability of the oral solution, were evaluated using descriptive statistics. The time to onset and the duration of the warming sensation were evaluated using Kaplan-Meier methodology. Time to onset was censored at 10 minutes if it had not occurred in the first 10 minutes after dosing. Duration of the warming sensation was censored at 10 minutes minus the time of onset if the end of the warming sensation had not occurred in the first 10 minutes after dosing. Adverse events were coded using the Medical Dictionary for Regulatory Activities (version 19.0) and evaluated descriptively in all subjects who received any dose of the study medication. Other safety parameters were also evaluated descriptively. 

## Results 

### Subjects 

A total of 58 subjects consented to study participation and were screened; of these, 57 subjects met all eligibility criteria and were enrolled. The remaining subject was not enrolled because he had a productive cough and sore throat that was rated as severe. All enrolled subjects completed the study. The study cohort had a mean age of 38.7 years and included 4 adolescents; all subjects were white, and a majority (57.9%) was female (Table 1). All subjects had mild or moderate productive cough at screening, consistent with the eligibility requirements. Other cold symptoms, when present, were generally described as mild to moderate in severity. 

### Assessment of the warming sensation 

**Figure 1.**
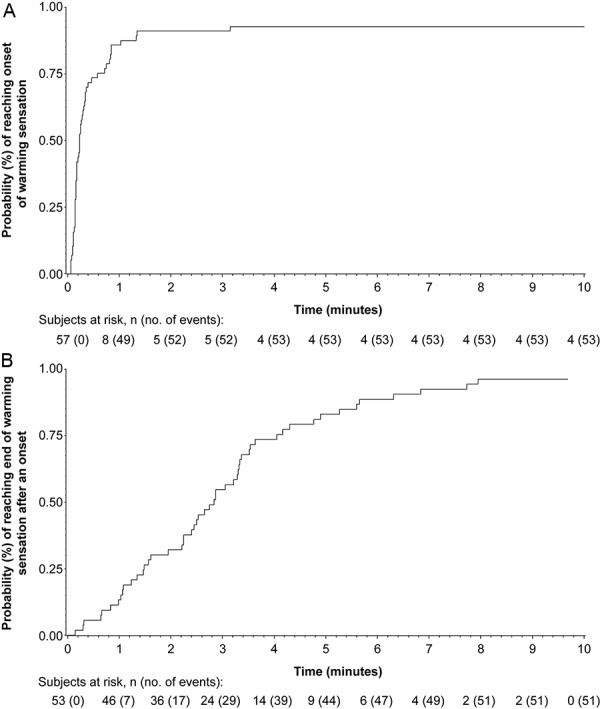
Time to onset (A) and duration (B) of the warming sensation following administration of the study medication (safety population).

The study medication was administered in a median of 6 seconds (range, 2 – 23 seconds; mean ± SD, 6.8 ± 4.1 seconds). Following administration of the study product, the intensity of the warming sensation increased from a mean of 6.8 mm on the VAS at baseline to a mean of 36.1 mm at 60 seconds postdose. The median intensity increased from 2.0 mm at baseline (range, 0 – 33 mm) to 30.0 mm at 60 seconds postdose (range, 0 – 89 mm). Of the 57 subjects, 53 (93.0%) reported the onset of a warming sensation within 10 minutes after administration of the study medication. The median time to onset of a warming sensation was ~ 14 seconds after administration (range, 4 seconds to 10 minutes) (Figure 1A). The duration of the warming sensation was evaluable in 51 of the 53 subjects with a reported onset, and it lasted for a median of 2.8 minutes (range: 9 seconds to 9.8 minutes after onset) (Figure 1B). In the assessment of the acceptability of the warming sensation, 30 subjects (52.6%) rated the warming sensation as “just about right (pleasant warming),” whereas 25 subjects (43.9%) considered the warming sensation to be either “too weak” (28.1%) or “much too weak” (15.8%). The remaining 2 subjects (3.5%) rated the warming sensation as “too strong.” 

### Overall opinion of the warming sensation and study medication 

Most subjects had a positive opinion of the warming sensation, with 33 subjects (57.9%) providing opinions of “like moderately” or better. Only 4 subjects (7.1%) provided opinions of “dislike moderately” or “dislike very much”, and no subject provided an opinion of “dislike extremely.” At the end of the 1-hour observation period, most subjects had an overall opinion of “fair” or “good” for the taste and texture of the oral solution (Table 2). In the overall assessment of the oral solution, 2 subjects (3.5%) provided a rating of “excellent,” 21 (36.8%) a rating of “good,” 29 (50.9%) a rating of “fair,” and 5 (8.8%) a rating of “poor” (Table 2). 

### Safety and tolerability 

The study medication was safe and generally well tolerated. None of the subjects reported any treatment-emergent adverse events, and changes in vital signs during the study were minimal and were considered by the investigator not to be clinically relevant. On oropharyngeal examination, 52 subjects (91.2%) had normal findings at screening and at the end of the study; none of the 5 subjects with abnormal results (which were present at baseline and unchanged at the end of the study) showed any signs of lesions or irritation of the oral mucosa following treatment. On physical examination, nearly all subjects had normal findings at each time point for all body systems assessed. Only 1 abnormal oral mucosa finding was considered clinically significant: red, hypertrophic, purulent tonsils in a subject who entered the study with ongoing tonsillitis. 

## Discussion 

This study was designed to evaluate perceptions and acceptability of the warming sensation of the flavoring agent IFF flavor 316282 when included in a 2% acetylcysteine oral solution for use by subjects with productive cough due to URTI. The majority of subjects (93.0%) perceived a warming sensation within 10 minutes of swallowing the oral solution, with a median time to onset of ~ 14 seconds and a median duration of ~ 2.8 minutes. Using a 100-mm VAS, the subjects indicated that the intensity of the warming sensation increased from baseline by a mean of 29.2 mm when evaluated at 60 seconds after taking the oral solution. Just over half of the subjects considered the warming sensation to be “just about right,” whereas most of the others thought the warming sensation was “too weak” or “much too weak.” Most also indicated that they liked the warming sensation, with only 7 subjects (12.4%) indicating that they disliked it even slightly. In previous studies of other cough and cold products containing IFF flavor 316282, a majority of subjects also indicated that the warming sensation was pleasantly warming, whereas a number of others would have preferred it to be stronger [5, 6]. Taken together, these data suggest that the warming sensation may be considered a desirable feature in a cough and cold product, perhaps contributing to a soothing sensation. When assessed at 1 hour after administration, most subjects found the taste and texture of the oral solution to be acceptable, and most provided an overall opinion assessment of “fair” (50.9%) or “good” (36.8%). 

No safety concerns related to the study product were identified. Treatment-emergent adverse events were not reported in any subjects, and any changes in vital signs were minimal and not clinically relevant. The safety and tolerability assessment included an oropharyngeal examination to determine if the oral solution was irritating to the oral mucosa. The majority of subjects (91.2%) had normal findings, and those with abnormal results at both baseline and the end of the study did not have any evidence of oral mucosal irritation or lesions. 

The main limitations of this study are its open-label design and lack of a control oral solution without IFF flavor 316282. However, it is difficult to provide appropriate controls in this type of study, in that removing the flavoring agent may make the solution less palatable, or substituting another flavoring agent without warming properties may alter its taste and thereby influence subject preferences. 

## Conclusion 

In summary, this study shows that the majority of subjects with a productive cough due to URTI perceive a pleasant warming sensation that occurs rapidly and is relatively short-lived following ingestion of a new 2% acetylcysteine oral solution containing IFF flavor 316282. Most subjects liked the warming sensation and found the study product to be acceptable overall and in terms of taste and texture. These findings suggest that the addition of IFF flavor 316282 to a 2% acetylcysteine oral solution may increase its acceptability, which, in turn, may result in improved compliance by subjects with productive cough using the product to relieve flu and cold symptoms. 

## Acknowledgment 

The authors thank Maria Anschütz for providing project management and Judith Debes, the coordinating study nurse, both of whom are affiliated with SocraTec R&D GmbH. 

Medical writing assistance was provided by Barry Weichman, PhD, and Kristin Larsen, PhD, of Peloton Advantage and was funded by GlaxoSmithKline Consumer Healthcare. GlaxoSmithKline Consumer Healthcare provided a full review of the article. 

## Funding 

This study was sponsored by GlaxoSmithKline Consumer Healthcare. 

## Conflict of interest 

Frank Donath and Lucy Shneyer report no conflicts of interest. 

Anna Grinienko, Pascal Mallefet, and Michel Jean-Pierre Ozun are employees of GlaxoSmithKline Consumer Healthcare. 

**Table 1.** Subject demographics and baseline characteristics (safety ­population).

Characteristic	N = 57
Age, years	
Mean (SD)	38.7 (13.88)
Range	13 – 73
Age group, n (%)	
12 to < 18 years	4 (7.0)
18 to < 35 years	20 (35.1)
35 to < 70 years	31 (54.4)
≥ 70 years	2 (3.5)
Sex, n (%)	
Male	24 (42.1)
Female	33 (57.9)
Race, n (%)	
White	57 (100)
Ethnicity, n (%)	
Not Hispanic or Latino	57 (100)
Weight, mean (SD), kg	77.3 (19.04)
Height, mean (SD), cm	172.6 (8.70)
Productive cough at screening, n (%)	
Mild	31 (54.4)
Moderate	26 (45.6)
Other cold symptoms, n (%)	
Rhinorrhea	50 (87.8)
Nasal congestion	40 (70.2)
Sore throat	40 (70.2)
Sneezing	34 (59.7)
Headache	26 (45.6)
Bodily pain	24 (42.1)
Fever	1 (1.8)

SD = standard deviation.

**Table 2.** Overall opinion of taste, texture, and oral solution at end of 1-hour evaluation period (safety population).

Rating, n (%)	Overall opinion of taste (N = 57)	Overall opinion of texture (N = 57)	Overall opinion of oral solution (N = 57)
Excellent	0 (0)	2 (3.5)	2 (3.5)
Good	22 (38.6)	26 (45.6)	21 (36.8)
Fair	23 (40.4)	27 (47.4)	29 (50.9)
Poor	11 (19.3)	1 (1.8)	5 (8.8)
Unacceptable	1 (1.8)	1 (1.8)	0 (0)
